# Independent validation of CT radiomics models in colorectal liver metastases: predicting local tumour progression after ablation

**DOI:** 10.1007/s00330-023-10417-5

**Published:** 2023-11-21

**Authors:** Denise J. van der Reijd, Corentin Guerendel, Femke C. R. Staal, Milou P. Busard, Mateus De Oliveira Taveira, Elisabeth G. Klompenhouwer, Koert F. D. Kuhlmann, Adriaan Moelker, Cornelis Verhoef, Martijn P. A. Starmans, Doenja M. J. Lambregts, Regina G. H. Beets-Tan, Sean Benson, Monique Maas

**Affiliations:** 1 Department of Radiology, Antoni Van Leeuwenhoek – The Netherlands Cancer Institute, Plesmanlaan 121, 1066 CX Amsterdam, The Netherlands; 2 GROW School for Oncology and Reproduction, Maastricht University, Universiteitssingel 40, 6229 ER Maastricht, The Netherlands; 3 Department of Surgery, Antoni Van Leeuwenhoek – The Netherlands Cancer Institute, Plesmanlaan 121, 1066 CX Amsterdam, The Netherlands; 4 Department of Radiology and Nuclear Medicine, Erasmus MC Cancer Institute, University Hospital Rotterdam, Dr. Molewaterplein 40, 3015 GD Rotterdam, The Netherlands; 5 Department of Surgical Oncology, Erasmus MC Cancer Institute, University Hospital Rotterdam, Dr. Molewaterplein 40, 3015 GD Rotterdam, The Netherlands; 6 Institute of Regional Health Research, University of Southern Denmark, Campusvej 55, DK 5230 Odense M, Denmark; 7 Department of Cardiology, Amsterdam University Medical Centers, University of Amsterdam, Meibergdreef 9, 1105 AZ Amsterdam, The Netherlands

**Keywords:** Colorectal cancer, Liver, Metastases, Machine learning, Validation study

## Abstract

**Objectives:**

Independent internal and external validation of three previously published CT-based radiomics models to predict local tumor progression (LTP) after thermal ablation of colorectal liver metastases (CRLM).

**Materials and methods:**

Patients with CRLM treated with thermal ablation were collected from two institutions to collect a new independent internal and external validation cohort. Ablation zones (AZ) were delineated on portal venous phase CT 2–8 weeks post-ablation. Radiomics features were extracted from the AZ and a 10 mm peri-ablational rim (PAR) of liver parenchyma around the AZ. Three previously published prediction models (clinical, radiomics, combined) were tested without retraining. LTP was defined as new tumor foci appearing next to the AZ up to 24 months post-ablation.

**Results:**

The internal cohort included 39 patients with 68 CRLM and the external cohort 52 patients with 78 CRLM. 34/146 CRLM developed LTP after a median follow-up of 24 months (range 5–139). The median time to LTP was 8 months (range 2–22). The combined clinical-radiomics model yielded a c-statistic of 0.47 (95%CI 0.30–0.64) in the internal cohort and 0.50 (95%CI 0.38–0.62) in the external cohort, compared to 0.78 (95%CI 0.65–0.87) in the previously published original cohort. The radiomics model yielded c-statistics of 0.46 (95%CI 0.29–0.63) and 0.39 (95%CI 0.28–0.52), and the clinical model 0.51 (95%CI 0.34–0.68) and 0.51 (95%CI 0.39–0.63) in the internal and external cohort, respectively.

**Conclusion:**

The previously published results for prediction of LTP after thermal ablation of CRLM using clinical and radiomics models were not reproducible in independent internal and external validation.

**Clinical relevance statement:**

Local tumour progression after thermal ablation of CRLM cannot yet be predicted with the use of CT radiomics of the ablation zone and peri-ablational rim. These results underline the importance of validation of radiomics results to test for reproducibility in independent cohorts.

**Key Points:**

*• Previous research suggests CT radiomics models have the potential to predict local tumour progression after thermal ablation in colorectal liver metastases, but independent validation is lacking.*

*• In internal and external validation, the previously published models were not able to predict local tumour progression after ablation.*

*• Radiomics prediction models should be investigated in independent validation cohorts to check for reproducibility.*

**Graphical Abstract:**

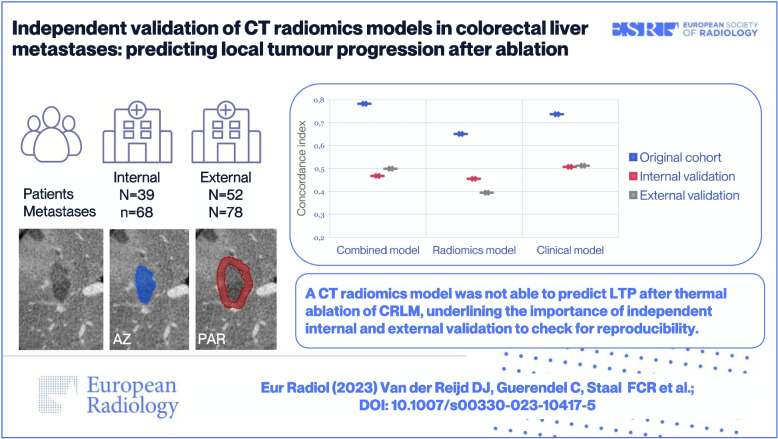

**Supplementary Information:**

The online version contains supplementary material available at 10.1007/s00330-023-10417-5.

## Introduction

The preferred treatment choice for colorectal liver metastases (CRLM) is resection, but not all metastases nor patients are eligible for resection. An alternative and complementary strategy is thermal ablation, including microwave ablation (MWA) and radiofrequency ablation (RFA) [1, 2]. After thermal ablation of CRLM, local tumour progression (LTP) rates of 6–46% have been reported [3–8]. LTP is defined as the recurrence of tumour foci at the edge of the ablation zone after initial follow-up imaging showing adequate ablation [9, 10]. The detection of LTP can be challenging since post-ablation effects and recurrent disease have comparable densities on contrast enhanced (ce) CT [11]. This results in a sensitivity of 53% for ceCT for the detection of LTP [4]. So to detect LTP, imaging at multiple subsequent time points may be necessary, consequently causing a delay in the detection and treatment of LTP.

To overcome this delay, we recently performed a study to predict LTP in CRLM with the use of radiomics of the post-ablation CT images [12]. If the prediction of LTP is successful, patients with a high risk for LTP can undergo complementary treatment without delay, and a de-intensified follow-up schedule can be considered for low-risk patients. In the previously published original study, we developed and compared three prediction models, including clinical parameters, radiomics features of both the ablation zone (AZ) and the peri-ablational rim (PAR), as well as a combination of clinical and radiomics parameters. The combined clinical-radiomics model yielded the highest performance with a concordance (c-) statistic of 0.78 (95% confidence interval (95%CI) 0.65–0.87). The performances were retrieved with leave-one-out cross-validation (LOOCV), i.e., the models were not validated on independent patient cohorts. To evaluate whether results can be applied to other populations, external validation is crucial [13]. Therefore, the aim of the current study is to validate the clinical-radiomics prediction models from the original study to predict LTP after thermal ablation of CRLM using both independent internal and external validation cohorts.

## Material and methods

### Patient selection

This multicentre retrospective study was approved by the Institutional Review Board of both institutions (IRBd18.066/MEC-2019–0850), and informed consent was waived. A data license agreement was established to transfer all data to the primary research centre. For the internal validation cohort, medical records were reviewed from April 2018 until August 2021 in the same institution (The Netherlands Cancer Institute Amsterdam) where the original study was performed. In the original study, patients were included up until April 2018. For the external validation cohort, medical records were searched from January 2007 until October 2019 in the second institution (Erasmus Medical Centre Rotterdam).

**Fig. 1:**
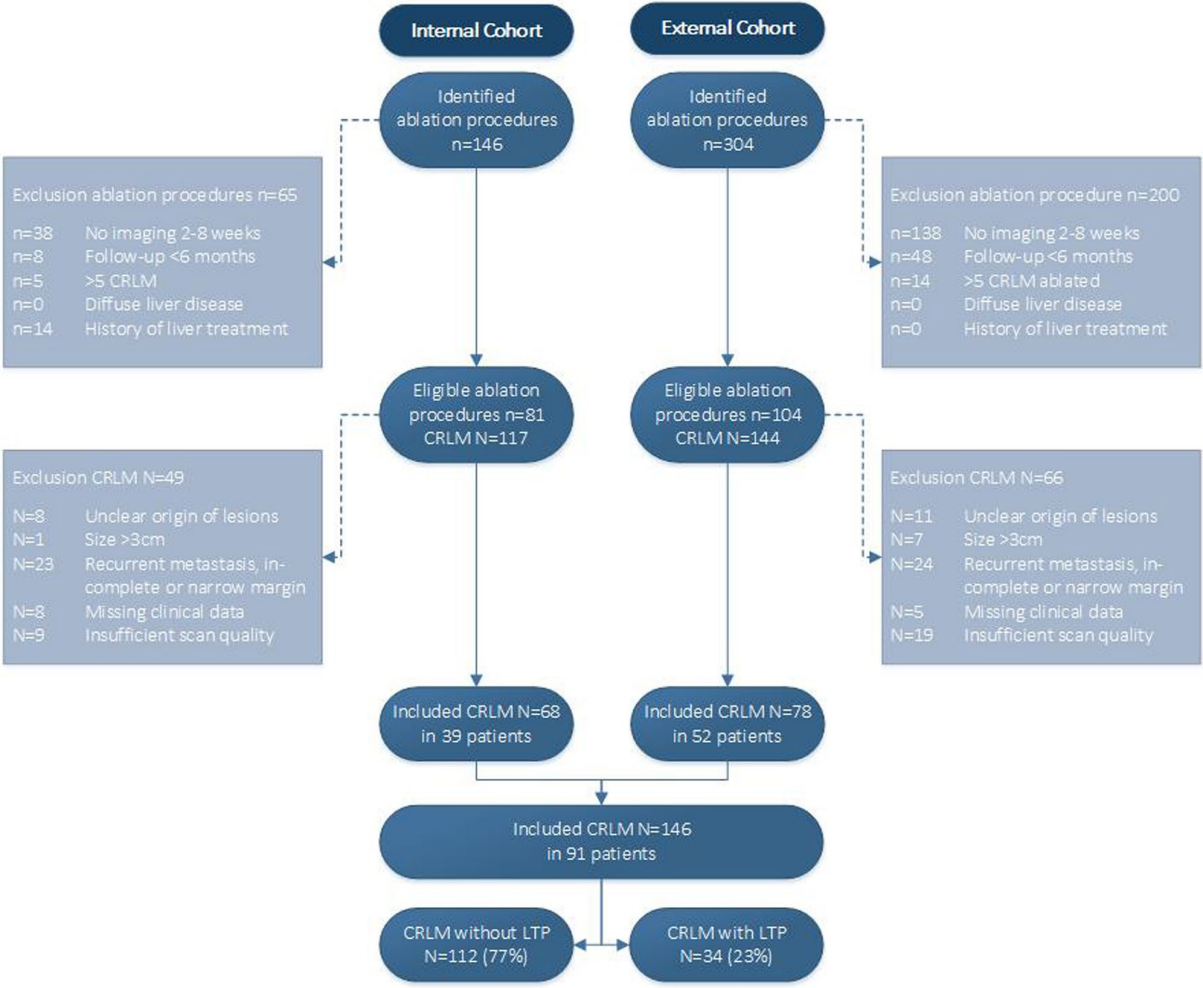
Flowchart of the patient selection process

The patient selection process was in line with the original study in order to select a comparable patient cohort. The original inclusion criteria comprised of (1) patients successfully treated with thermal ablation for CRLM; (2) histopathological confirmation of CRLM; (3) portal venous phase (PVP) CT available 2–8 weeks after ablation. The exclusion criteria were (1) < 6 months of follow-up without LTP; (2) > 5 CRLM; (3) unclear origin of liver metastases; (4) ablated CRLM of size > 3 cm; (5) history of diffuse liver disease; (6) history of liver treatment which could affect the parenchyma (such as stereotactic body radiation therapy (SBRT), portal vein embolisation (PVE), transarterial chemoembolisation (TACE)); (6) incomplete ablation (including residual disease, ablation margins < 5 mm and re-ablations); (7) missing clinical data (e.g. no pre-ablation imaging available); (8) delineation problems including artefacts, air or abscess within the AZ and insufficient scan quality. Due to a relatively short inclusion period compared to the external and original cohorts, the number of eligible patients for the internal validation was small. Hence, to increase the sample size for the internal cohort, the exclusion criterion ‘ > 5 CRLM’ was changed into ‘ > 5 CRLM ablated’. This adjustment was deemed not to influence the results, since it was made under the assumption that the AZ texture is not correlated with the number of CRLM present in one liver. A flowchart of the patient selection process is depicted in Fig. 1. Patient characteristics were collected from the medical records and are presented per cohort in Table 1.

**Table 1:** Patient and lesion characteristics

Patient characteristic	Internal cohort	External cohort	Original cohort	*p-value* * ≤ 0.05
Total number of patients	*n* = 39	*n* = 52	*n* = 82	
Mean age (y) at time of ablation ± SD	61 ± 11	66 ± 11	63 ± 10	0.047*
Sex (%)				0.21
Female	14 (36)	15 (29)	36 (44)	
Male	25 (64)	37 (71)	46 (56)	
Timing of metastases (%)				< 0.01*
Synchronous	31 (79)	40 (77)	45 (55)	
Metachronous	8 (21)	12 (23)	37 (45)	
Location colon primary tumour (%)				0.93
Right	9 (23)	12 (23)	17 (32)	
Left	30 (77)	40 (77)	65 (68)	
T-stage (%)				0.31
T1−2	5 (13)	9 (17)	7 (9)	
T3−4	34 (87)	43 (83)	75 (91)	
N-stage (%)				0.08
N0	16 (41)	24 (46)	23 (28)	
N +	23 (59)	28 (54)	59 (72)	
Chemotherapy (%)				0.28
No adjuvant CTx	18 (46)	32 (62)	41 (50)	
Adjuvant CTx	21 (54)	20 (38)	41 (50)	
Lesion characteristics				
Total number of ablated CRLM	*N* = 68	*N* = 78	*N* = 127	
Mean number of ablated CRLM per patient ± SD	2.2 ± 1.0	2.0 ± 1.2	2.1 ± 1.2	0.21
Mean size of ablated CRLM ± SD (mm)	11 ± 7	13 ± 7	18 ± 6	0.047*
Mean number of CRLM pre-ablation ± SD	3.7 ± 2.2	2.7 ± 1.5	2.7 ± 1.4	< 0.01*
Median follow-up (m) (range)	24 (5–50)	24 (5–139)	24 (6–115)	0.91
Number of lesions with LTP (%)	11 (16)	23 (29)	33 (26)	0.15
Median time to LTP (m) (range)	8 (2–22)	10 (2–21)	6 (2–14)	0.10
Procedure location (%)				0.06
Open (operating room)	47 (69)	39 (50)	74 (58)	
Percutaneous (CT room)	21 (31)	39 (50)	53 (42)	
Technique used (%)				< 0.01*
RFA	0 (0)	68 (87)	101 (80)	
MWA	68 (100)	10 (13)	26 (20)	

Abbreviations: *CRLM* colorectal liver metastases, *CT* computed tomography, *CTx* chemotherapy treatment, *LTP* local tumour progression,* m* months, *MWA* microwave ablation, *RFA* radiofrequency ablation, *SD* standard deviation, *y* years

### Ablation procedures

Ablation procedures were performed either percutaneously under CT or ultrasound guidance or open, guided by intraoperative ultrasound. All percutaneous ablations were performed by an interventional radiologist under sedation analgesia, epidural, or general anaesthesia. The open ablations were performed under general anaesthesia by a liver surgeon, either with or without the assistance of an interventional radiologist. The choice between RFA and MWA was based on the availability and physician’s preferences. Three different systems were used for RFA: the Cool-tip™ RF Ablation System E Series (Medtronic), the StartBurst® Radiofrequency Ablation system (AngioDynamics), and the AMICA Microwave and RF system (HS Hospital Service). For MWA, the NeuWave™ Microwave Ablation System of Ethicon (Johnson&Johnson), the Emprint™ Ablation System with Thermosphere™ Technology (Medtronic), and the AMICA Microwave and RF system (HS Hospital Service) were used. Procedures were carried out in accordance with the CIRSE Standards of Practice [10].

### CT image acquisition

Contrast enhanced CT image acquisition was performed on a total of 19 different CT scanners. Intravenous contrast was injected at a rate of 3 ml/s followed by a 30 ml saline flush. Both bolus triggering software and fixed delay times (70 s post-injection for PVP) were used, depending on the CT scanner. Detailed information on scanning parameters is displayed in Table 2.

**Table 2:** Scanning parameters

Parameters	Internal cohort	External cohort	Original cohort
CT manufacturers	Philips, Siemens, Toshiba	Siemens	Philips, Siemens, Toshiba
CT models*	7^a^	11^b^	4^c^
Median kVp (range)	120 (80–120)	120 (90–120)	120 (100–135)
Median X-ray tube current in mA	223 (150–637)	265 (56–743)	262 (70–494)
Mean slice thickness in mm (SD)	1.3 ± 0.4	3.3 ± 1.0	2.0 ± 1.4
Contrast agent	Omnipaque 300	Visipaque 320	Omnipaque 300

Abbreviations: *kVp* kilovoltage peak, *mA* milliamperes, *mm* millimetre, *ms* milliseconds, *SD* standard deviation

^*^CT models specified per cohort: ^a^ Phillips Gemini TF 16, Siemens SOMATOM Sensation Open, Toshiba Aquilion, Phillips Vereos Digital, Siemens SOMATOM Confidence® RT Pro, Siemens SOMATOM Force, Siemens go.Open Pro. ^b^ Siemens Biograph mCT 128, Siemens SOMATOM Definition AS + , Siemens SOMATOM Definition Edge, Siemens SOMATOM Definition Flash, Siemens SOMATOM Drive, Siemens SOMATOM Force, Siemens SOMATOM Edge Plus, Siemens SOMATOM Sensation Open, Siemens SOMATOM Sensation 64, Siemens SOMATOM Sensation 16. ^c^ Phillips Gemini TF 16, Siemens SOMATOM Sensation Open, Toshiba Aquillion, Siemens SOMATOM Definition Edge

### Standard of reference to establish LTP

LTP was defined as any new tumour foci occurring in a 10 mm vicinity of the AZ on follow-up imaging within 24 months after thermal ablation [9]. Lesions were categorised as no LTP if the patient developed (1) no new CRLM; (2) new CRLM > 10 mm distance to the AZ; or (3) new CRLM within 10 mm of the AZ after > 24 months. Follow-up imaging consisted of regular follow-up ceCT, scheduled every 3 months in the first year, and 6 monthly thereafter until 5 years after ablation. In case of doubt, magnetic resonance imaging (MRI) or positron emission tomography (PET)-CT was used as a problem-solver. All liver imaging until the end of follow-up was checked for disease progression.

### Delineation and radiomics features

**Fig. 2:**
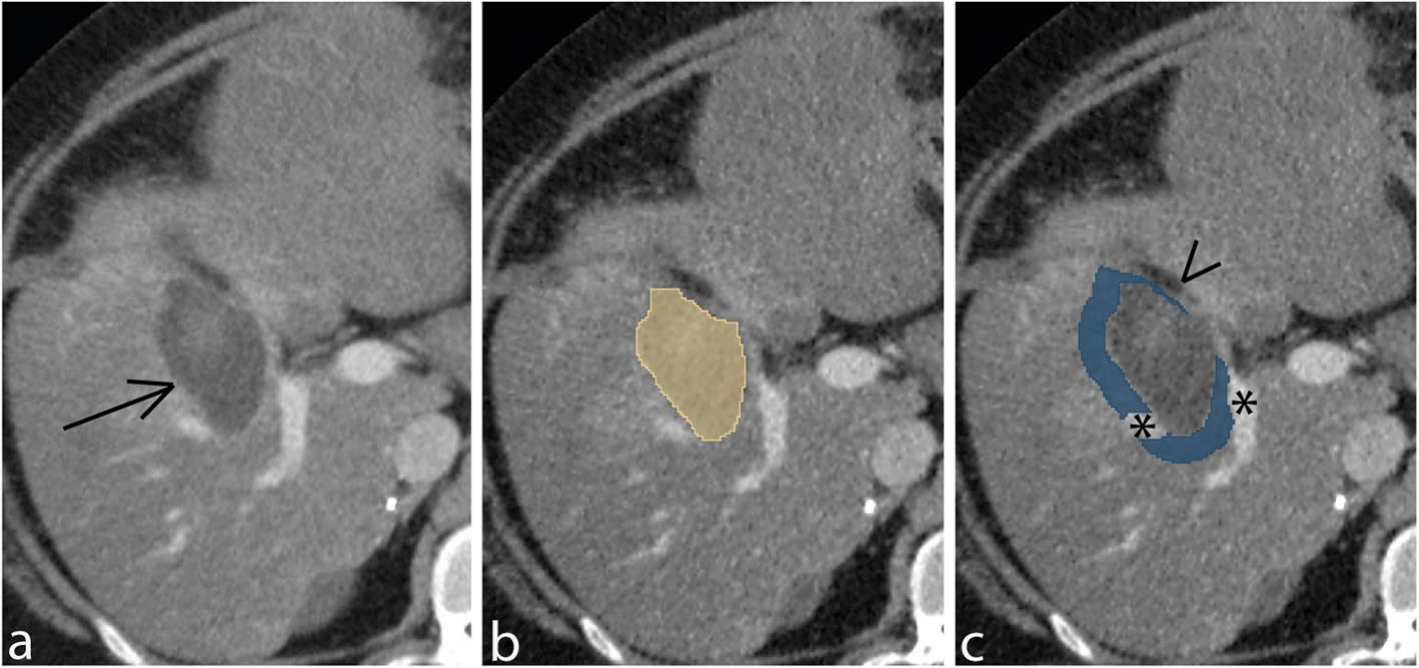
Delineation example. Post-ablation ceCT images of **a** the ablation zone (arrow), **b** the delineation of the ablation zone, and **c** the peri-ablational rim with the exclusion of the needle track ( <) and large vessels (*)

The manual delineations, pre-processing steps, and features extraction process were similar to the original study [12]. An example of the delineations is displayed in Fig. 2.

### Prediction models and analysis

**Fig. 3:**
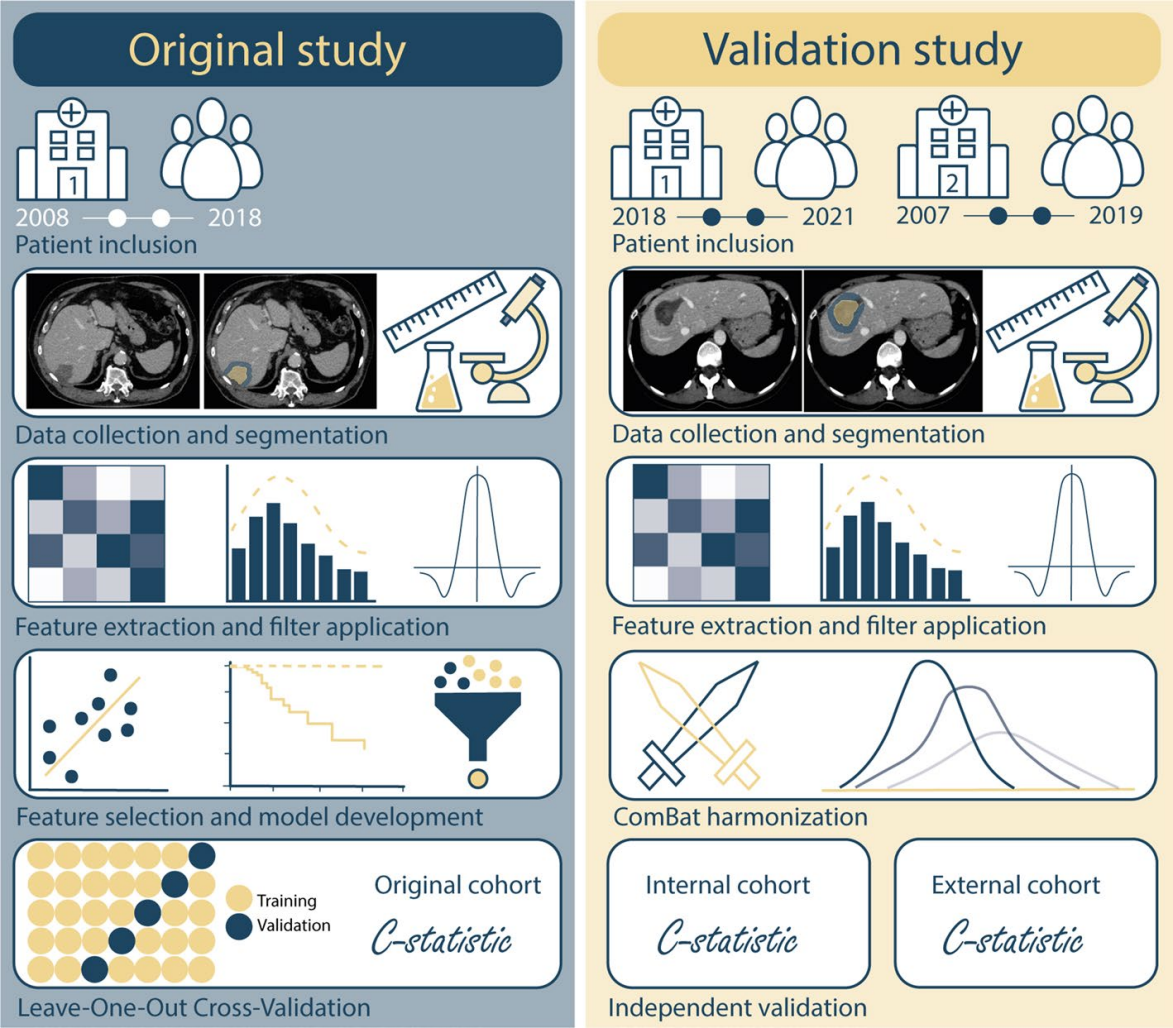
Methodology**.** Schematic presentation of the methodology of the current study (right) and the original study (left)

Baseline patient characteristics were compared between the cohorts, using the Kruskal Wallis test and chi-square test. *p* values ≤ 0.05 were considered statistically significant. The included features per model are presented in Table 3. For the two validation cohorts, the discriminative power of all three models was assessed using the c-statistic. ComBat harmonisation was applied to the radiomics features to harmonise between the three cohorts [14]. All statistical analyses were performed using RStudio software v1.4.1103. To assess the quality of this study, the Radiomics Quality Score (RQS) was calculated [15]. The methods of this study and the original study are schematically presented in Fig. 3.

**Table 3:** Included features per model

	Clinical model	Radiomics model	Combined model
Clinical features	Size T-stage Adjuvant chemotherapy	-	Size T-stage Adjuvant chemotherapy
Radiomics features	-	AZ_Uniformity_LoG-1.5 AZ_Variance_LoG-1.5 PAR_Uniformity_LoG-1.5 PAR_Variance_LoG-1.5	AZ_Uniformity_LoG-1.5 AZ_Skewness_original PAR_Uniformity_LoG-1.5 PAR_Mean_LoG-0.5 PAR_Skewness_LoG-0.5

Abbreviations: *AZ* ablation zone, *PAR* peri-ablational rim, *LoG* Laplacian of Gaussian filter

## Results

### Patient and lesion characteristics

The internal validation cohort included 68 CRLM in 39 patients. LTP was found in 11/68 CRLM (16%). The median time to LTP was 8 months (range 2–22), and the median follow-up for CRLM without LTP was 25 months (range 8–50). The external cohort comprised of 78 CRLM in 52 patients. Twenty-three out of 78 CRLM (29%) developed LTP with a median time to LTP of 10 months (range 2–22 months). The CRLM without LTP had a median follow-up of 29 months (range 6–139). The median ablation to CT interval was 31 days (range 14–50, IQR 24–44 days) and 42 days (range 14–56, IQR 20–48 days) for the internal and external cohort, respectively. Patient and lesion characteristics were similar in terms of sex, primary tumour characteristics, and chemotherapy treatment. A higher mean age (66 vs 61 and 63) was found in the external validation cohort (*p* = 0.047). Larger CRLM were ablated (*p* = 0.047) in the original cohort (18 ± 6), compared to the internal (11 ± 7 mm) and external cohorts (13 ± 7). Significantly more metachronous metastases were included in the validation cohorts compared to the original cohort (21 and 23% vs 45%, *p* < 0.01). Lastly, all CRLM (100%) were treated with MWA in the internal cohort, while the majority were treated with RFA in the original and external cohorts (80% and 87%, respectively, *p* < 0.01).

### Model performance

For the internal validation cohort, a c-statistic of 0.47 (95%CI 0.30–0.64) was found for the combined model. The radiomics model showed a c-statistic of 0.46 (95%CI 0.29–0.63) and the clinical model 0.51 (95%CI 0.34–0.68). In external validation, the combined model yielded a c-statistic of 0.50 (95%CI 0.38–0.62), the radiomics model 0.40 (95%CI 0.28–0.52), and the clinical model 0.51 (95%CI 0.39–0.63). ComBat harmonisation yielded no improvement in the combined or radiomics models. Results are presented in Table 4. This study reached an RQS of 50%. The distribution of RQS points is displayed in Supplementary Table 1.

**Table 4:** Model performances

Model	Internal cohort C (95%-CI)	External cohort C (95%-CI)	Original cohort LOOCV C (95%-CI)
Combined model	0.467 (0.301–0.640)	0.499 (0.378–0.621)	0.783 (0.648–0.871)
Radiomics model	0.455 (0.291–0.630)	0.394 (0.279–0.522)	0.651 (0.519–0.830)
Clinical model	0.507 (0.336–0.676)	0.512 (0.389–0.632)	0.737 (0.578–0.837)
Combined model*	0.465 (0.299–0.639)	0.498 (0.377–0.620)	ref
Radiomics model*	0.455 (0.291–0.630)	0.394 (0.279–0.522)	ref

^*^ After ComBat harmonisation

Abbreviations: *C* concordance statistic, *CI* confidence interval, *LOOCV* leave-one-out-cross-validation, *ref* reference batch

## Discussion

This study evaluated the reproducibility of three previously published clinical-radiomics models to predict LTP after thermal ablation of CRLM. The models were validated in an independent internal and external validation cohort, and poor performances were found (C-statistics 0.40–0.51). The poor validation performance is most probably explained by overfitting: the models were trained too specifically for the training data and probably (also) used image noise or random fluctuations instead of true differences between the studied groups [16, 17]. In the original study, LOOCV was applied after model development. However, this is rather a test of the fit of the training data than of the quality of the model, which can result in an overoptimistic estimate of the performance [18].

We hypothesise our radiomics models overfitted on image noise caused by acquisition differences. Multiple studies show that acquisition parameters affect the values of the radiomics features [19–23]. Our cohorts were heterogeneous in terms of CT acquisition parameters, with 19 different CT scanners involved in validation and 5 scanners in the original study. In an attempt to account for the variability between scanners, we applied ComBat harmonisation to the three cohorts. The features were only marginally adjusted without a relevant effect on the performance, possibly because each batch already included multiple scanners. Preferably, the radiomics features would have been harmonised per CT scanner, but the number of patients allocated per batch was insufficient to allow for such harmonisation. Other acquisition differences were less likely to contribute to the low validation performance, such as the difference in iodine concentration per contrast agent or the tube current and voltage [23]. The differences in slice thickness were corrected by image resampling. Furthermore, additional steps, such as testing the intra-observer correlation of the segmentations or harmonising the features across scanners, could have been undertaken to enhance the reproducibility during model development.

Clinical heterogeneity between the cohorts might have contributed to the failure of the clinical model in validation. Despite the similar selection methodology, differences may have occurred due to (1) variations in hospital protocols and (2) adjustments over time due to treatment and scanner development. Both centres follow the Dutch clinical guidelines on the treatment of CRLM, but still, hospital variation occurs [24]. Especially, the eligibility of patients for thermal ablation based on ‘CRLM size’ and ‘number of CRLM ablated’ has evolved over the years. The use of MWA has rapidly increased over the last years, which resulted in technique differences between the cohorts. However, we do not think this is the reason for the low validation performance since the original study showed that the ablation technique did not significantly influence the radiomics features [12]. Moreover, two out of three parameters in the clinical model were ‘patient-specific’ (adjuvant chemotherapy and T-stage), while the prediction of LTP is a ‘lesion-specific’ outcome. A study exploring the risk factors for LTP found only ‘lesion-specific’ parameters were associated with LTP, and none of the ‘patient-specific’ parameters investigated were predictive for LTP [25]. This raises the question of how robust ‘patient-specific’ characteristics can be for the prediction of a ‘lesion-specific’ outcome.

Our study has several limitations. Firstly, the study design was retrospective and included a relatively small sample. Secondly, the LTP rates in our study were relatively high, which could be attributed to the long inclusion period, considering LTP rates were higher 15 years ago. The diagnosis of LTP was based on imaging, and the absence of histopathological evaluation could be considered a limitation, but it resembles how LTP is detected in clinical practice. Next, the minimum follow-up period of 6 months might have resulted in a small subset of patients being allocated to the wrong outcome group, given the median time to LTP of 8 months. Lastly, an arbitrary cut-off of 24 months was applied for the detection of LTP, as LTP after 24 months is rare and possibly involves new metastases rather than residual tumour clusters.

Due to the risk of overfitting the original model, we cannot draw any conclusions on the feasibility of LTP prediction based on CT radiomics. This study emphasises the need to assess the reproducibility of radiomics prediction models in independent patient cohorts. It underlines that no definite conclusions can be drawn from studies without proper internal and external validation. Future research aiming to explore radiomics in a similar setting should strive to minimise heterogeneity between and within patients’ cohorts, both in terms of clinical differences and imaging acquisition.

### Supplementary Information

Below is the link to the electronic supplementary material.

Supplementary file1 (PDF 111 KB)

## Abbreviations

AZ: Ablation zone
ceCT: Contrast enhanced computed tomography
CRLM: Colorectal liver metastases
c-statistic: Concordance statistic
LOOCV: Leave-one-out cross-validation
LTP: Local tumour progression
MRI: Magnetic resonance imaging
MWA: Microwave ablation
PAR: Peri-ablational rim
PET: Positron emission tomography
PVP: Portal venous phase
RFA: Radiofrequency ablation
RQS: Radiomics quality score

## Declarations

### Guarantor

The scientific guarantor of this publication is Monique Maas.

### Statistics and biometry

S. Roberti kindly (PhD Candidate at the Department of Epidemiology and Biostatistics, Antoni van Leeuwenhoek - The Netherlands Cancer Institute) provided statistical advice for this manuscript.

### Study subjects or cohorts overlap

No study subjects or cohorts have been previously reported.

### Methodology

• retrospective

• diagnostic study

• multicentre study
