# Supplementary material for: Independent validation of CT radiomics models in colorectal liver metastases: predicting local tumour progression after ablation
Source: Eur Radiol. 2023 Nov 21;34(6):3635–43. doi: 10.1007/s00330-023-10417-5 (PMC11166748; doi:10.1007/s00330-023-10417-5)
Supplement: Supplementary file 1 — Supplementary file1 (PDF 111 KB) [file 330_2023_10417_MOESM1_ESM.pdf]

Independent validation of CT radiomics models in colorectal liver metastases: predicting local tumour progression after ablation

Electronic Supplementary Material

Supplementary Table 1. Radiomics Quality Score

Criteria	Maximum	Our study
Image protocol quality, well-documented image protocols and/or usage of public image protocols	+2	+1
Multiple segmentations	+1	-
Phantom study on all scanners	+1	-
Imaging of individuals at multiple time points	+1	-
Feature reduction or adjustment for multiple testing	+3	+3
Multivariable analysis with non-radiomics features	+1	+1
Detect and discuss biological correlates	+1	+1
Cut-off analysis	+1	-
Discrimination statistics and statistical significance	+2	+2
Calibration statistics and statistical significance	+2	-
Prospective study	+7	-
Validation without retraining and without adaption of cut-off values	+5	+4
Comparison to ‘gold standard’	+2	+2
Potential clinical utility	+2	+2
Cost-effectiveness analysis	+1	-
Open science and data*	-	-
Total	32	16 (50%)

* Open science and data can only be evaluated after publication and the points are therefore excluded from the total score.
